# Supplementary material for: Molecular basis for redox control by the human cystine/glutamate antiporter system xc−
Source: Nat Commun. 2021 Dec 8;12:7147. doi: 10.1038/s41467-021-27414-1 (PMC8654953; doi:10.1038/s41467-021-27414-1)
Supplement: Supplementary file 1 — Supplementary Information [file 41467_2021_27414_MOESM1_ESM.pdf]

Molecular basis for redox control by the human cystine/glutamate antiporter System xc-.

Joanne L. Parker[1,*,#], Justin C. Deme[2,3,4#], Dimitrios Kolokouris[1], Gabriel Kuteyi[1], Philip C. Biggin[1], Susan M. Lea[2,3,4*], Simon Newstead[1,5*].

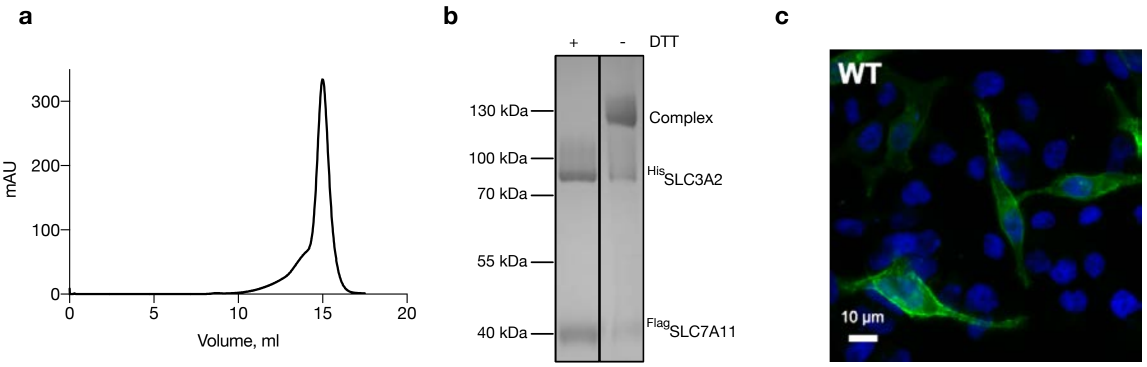

Supplementary Fig. 1 **a**, representative gel filtration trace of SLC7A11/SLC3A2 in LMNG:CHS. **b**, SDS-PAGE analysis of the purified complex in the presence or absence of 1 mM DTT, which dissociates the complex. **c**, Overexpressed GFP tagged SLC7A11 in the presence of SLC3A2 in HeLa cells is targeted mainly to the plasma membrane, Green SLC7A11-GFP, Blue DAPI.

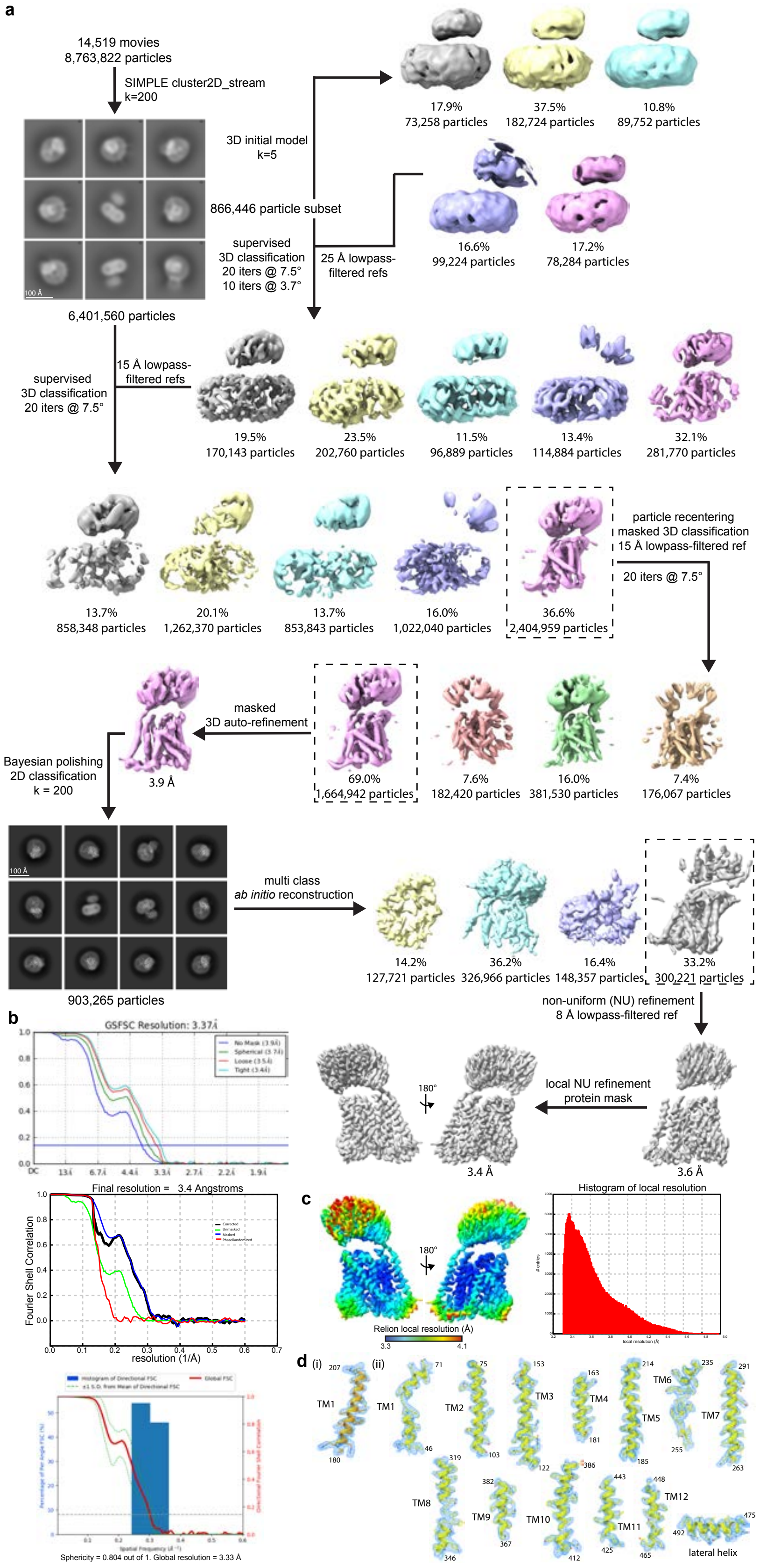

Supplementary Fig. 2. Cryo-EM processing workflow and local/global map quality for System xc-. a, Image processing workflow for System X_C^-. **b,** Gold-standard Fourier Shell

Correlation (FSC) curves used for global resolution estimates within cryoSPARC (top), RELION (middle), or 3DFSC (bottom). **c,** Local resolution estimation of reconstructed map as determined within RELION. Detergent density omitted for clarity. **d,** Close-up view of map and side-chain density for transmembrane helices and lateral helix. Volume contoured at threshold level of 0.25-0.3.

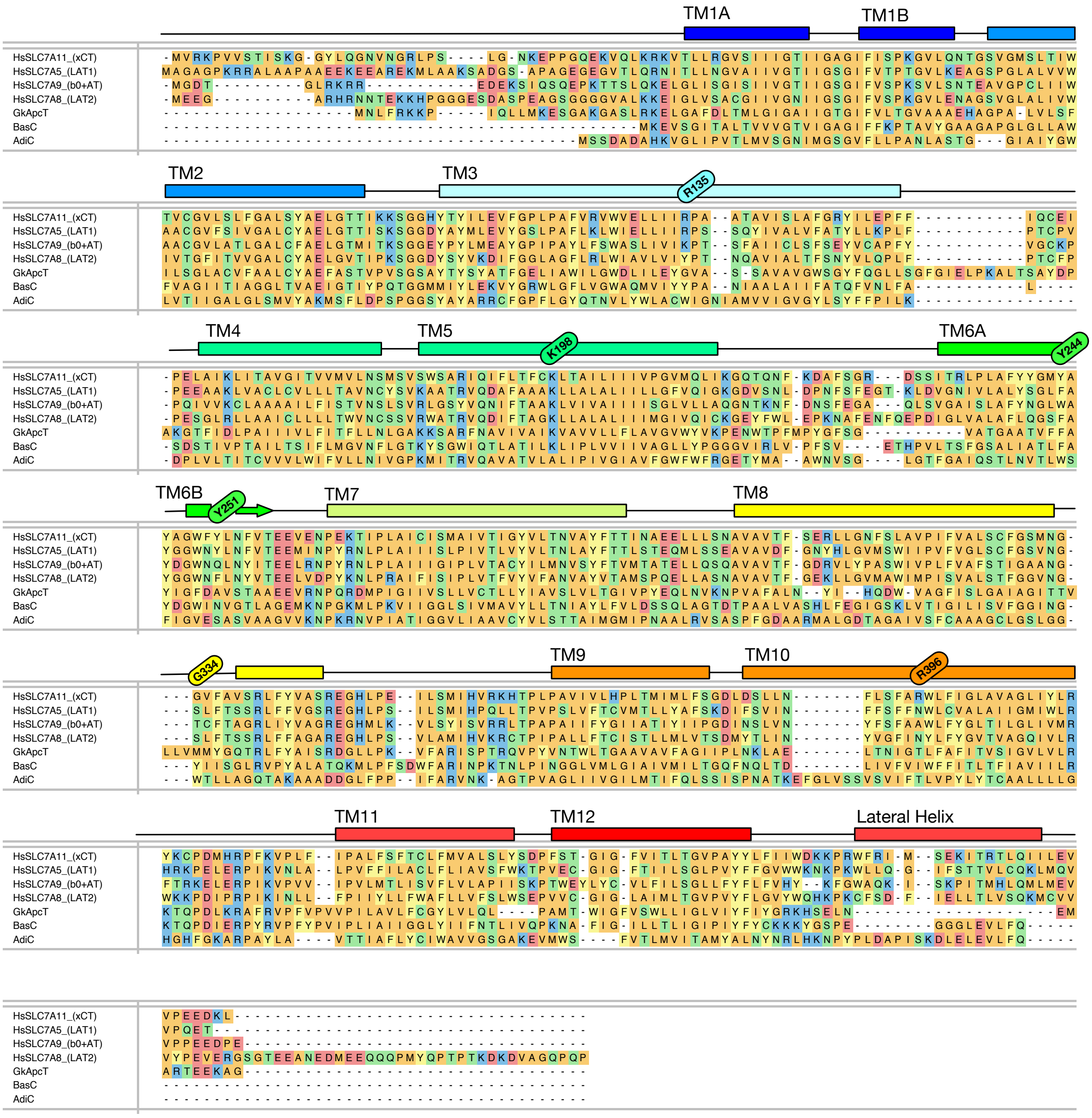

Supplementary Fig. 3. Sequence alignment of eukaryotic and prokaryotic SLC7 family members with experimentally determined structures. Residues with functional importance are highlighted as coloured ovals. Residues are coloured according to chemistry (Yellow=Aromatic; Red=Negatively charged; Blue=Positively charged; Orange=Nonpolar; Green=Polar). Secondary structure (alpha helices=rectangles; arrows=beta-sheet) is shown above for human xCT.

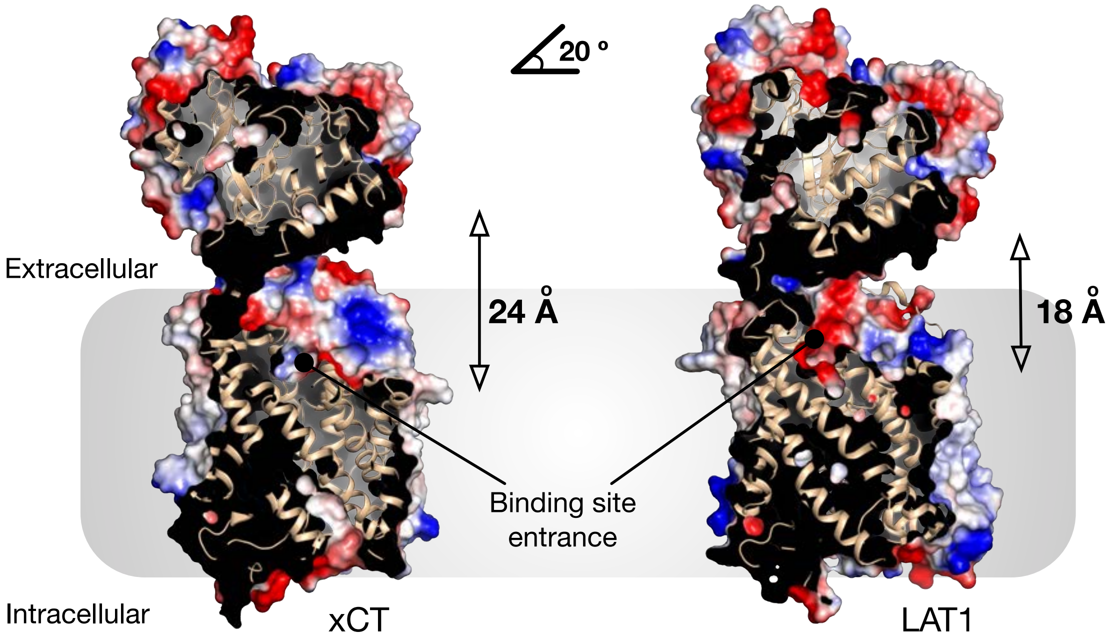

Supplementary Fig. 4. Structural comparison of the ectodomain of 4F2hc. The ectodomain is tilted approximately 20 ° away from the transporter domain relative to the position in LAT1, resulting in a wide entrance vestibule to the binding site.

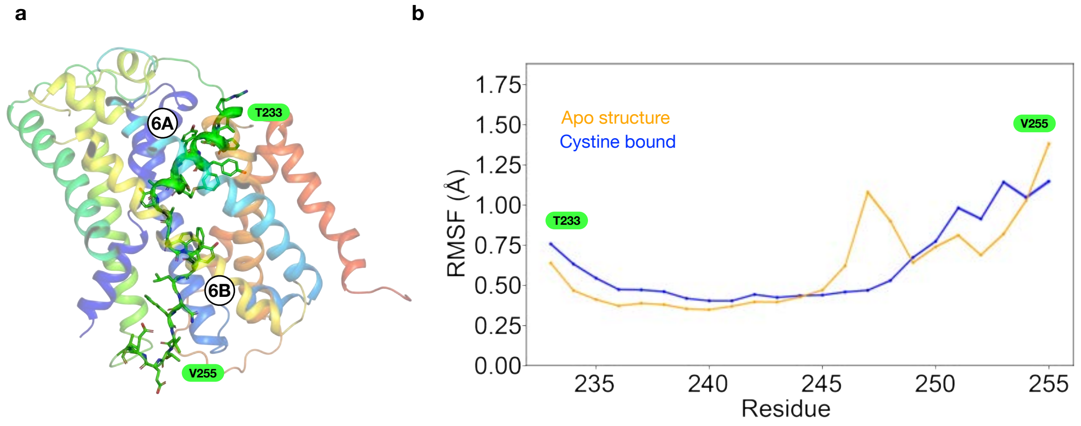

Supplementary Fig. 5. Analysis of TM6 stability using molecular dynamics. a, Cartoon highlighting the unusual conformation of TM6B. **b,** Comparison of root mean squared fluctuation (RMSF) for TM6 for simulations of the Apo (orange line) and Cystine-bound (blue line).

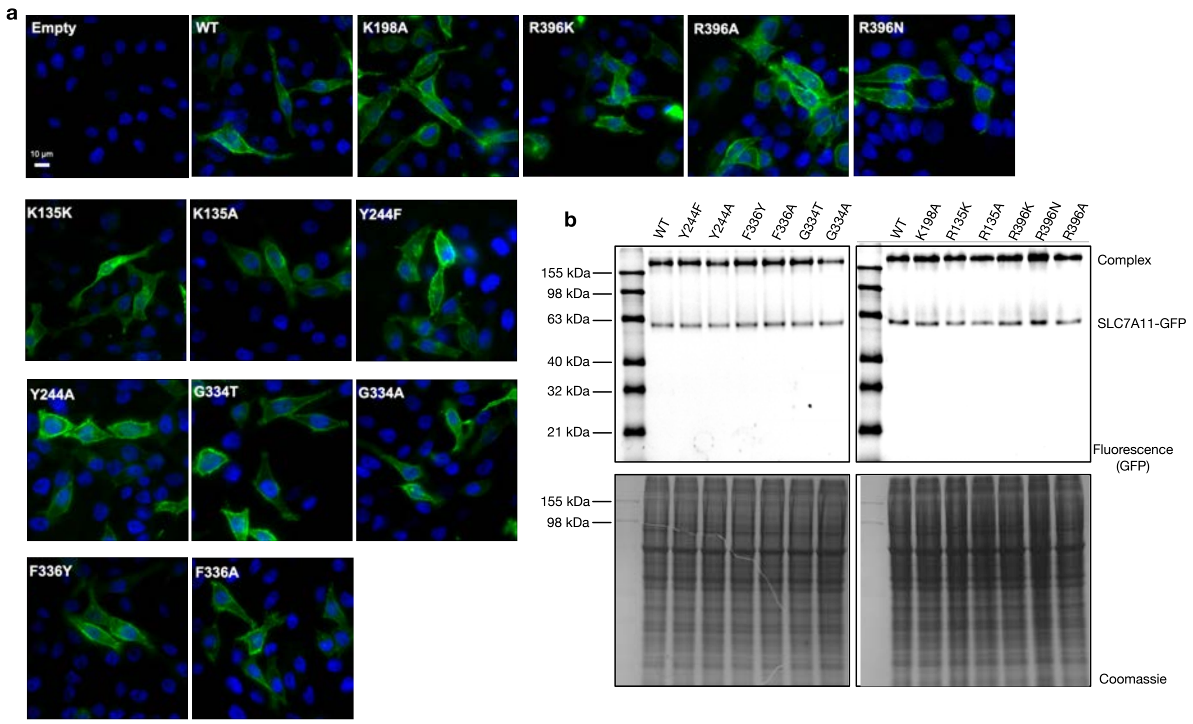

Supplementary Fig. 6. Expression analysis of the SLC7A11 mutant variants. a, Overexpression of the mutant variants of SLC7A11 with a C-terminal GFP tag indicates that all variants are targeted to the plasma membrane, similarly to WT. Green SLC7A11-GFP, Blue DAPI. **b**. In gel fluorescence of C-terminal tagged GFP variants show that all variants are produced in cells to a comparable level (upper panel Fluorescence) (lower panel Coomassie stained gels).

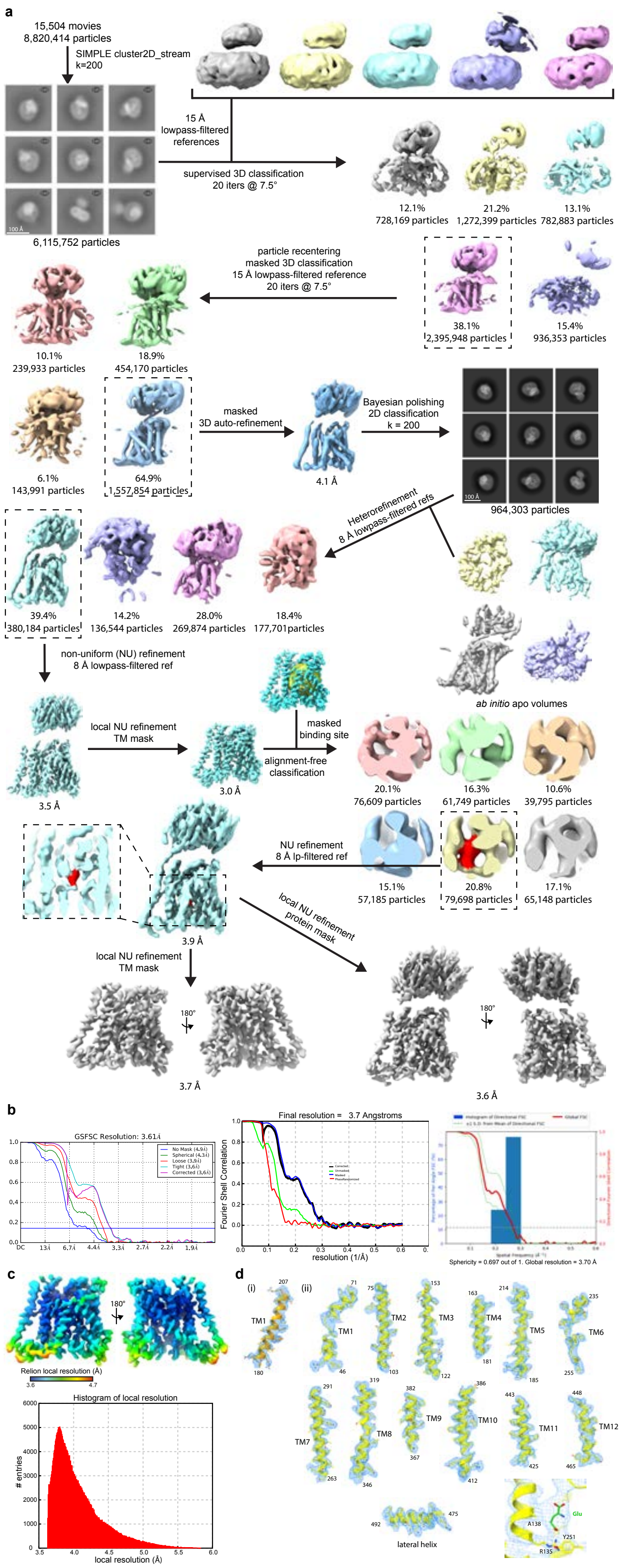

Supplementary Fig. 7. Cryo-EM processing workflow and local/global map quality for System xc- in complex with glutamate. a, Image processing workflow for System xc- in

complex with glutamate. **b,** Gold-standard Fourier Shell Correlation (FSC) curves used for global resolution estimates within cryoSPARC (left), RELION (middle), or 3DFSC (right). **c,** Local resolution estimation of focused refinement as determined within RELION. Detergent density omitted for clarity. **d,** Close-up view of map and side-chain density for transmembrane helices, lateral helix, and bound glutamate. Helices were contoured at threshold level of 0.17-0.3 and glutamate at threshold level of 0.34.

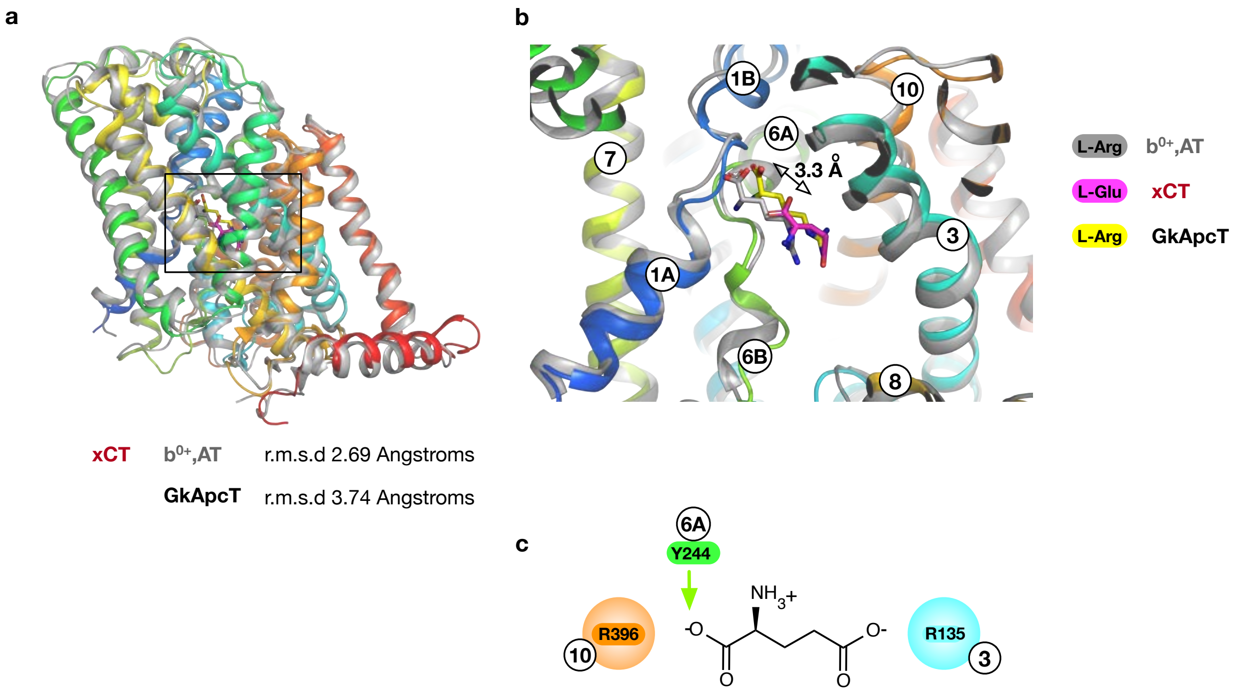

Supplementary Fig. 8. Comparison of the L-glutamate binding pose in xCT. a. Structural overlay of the xCT-glutamate complex with $b^{0+,}$AT in complex with L-Arginine (PDB:6lid) and GkApcT in complex with L-Arginine (PDB:6f34). For clarity the GkApcT backbone has been omitted. The r.m.s.d. values are given for these structures overlaid onto xCT (PDB:7P9U). **b.** Zoomed in view of the binding site showing the location of L-Arginine in $b^{0+,}$AT and GkApcT compared to xCT. **c.** Schematic of glutamate binding interactions, arrows indicate direction of the hydrogen bond. Key amino acids are labelled with the TM also indicated.

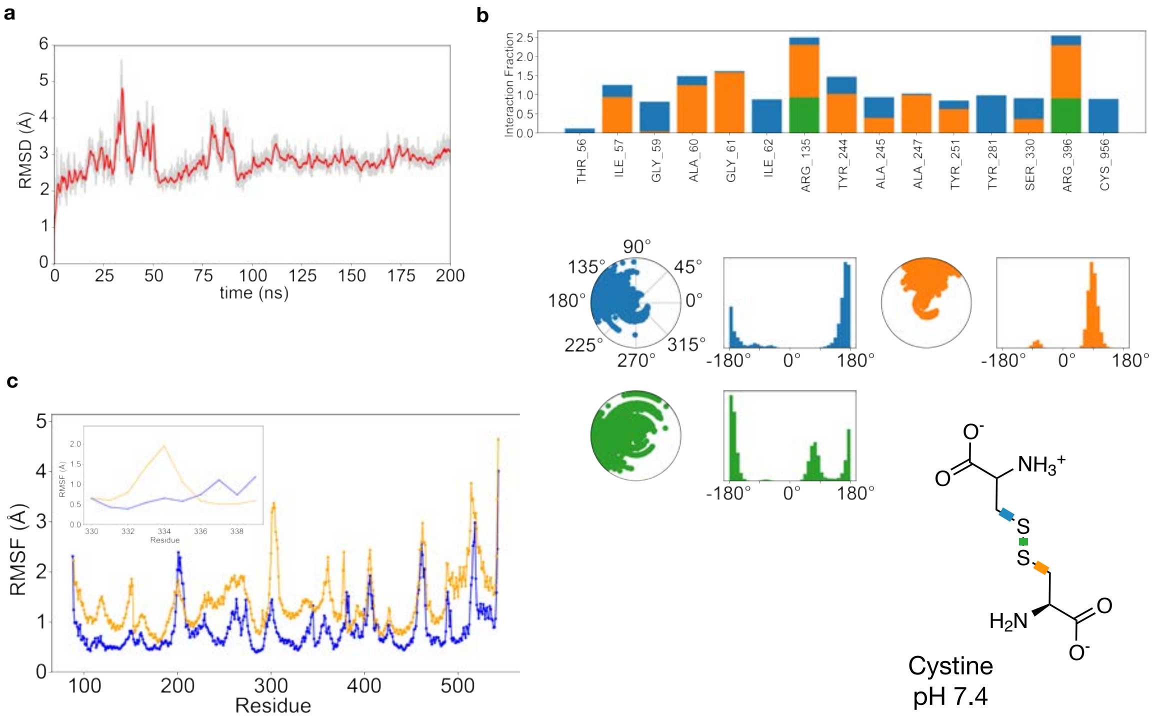

Supplementary Fig. 9. Molecular Dynamics (MD) simulation analysis of xCT with L-cystine. **a,** Root-mean-square deviation (in Å) of apo- xCT backbone atoms upon a least squares fit from a 200 ns simulation of the xCT-4F2hc heterodimer (grey: single data point, red: rolling average of 100 data points). The structure reaches an equilibrium plateau meaning the structure is stable and fit for MD simulation. **b,** Residue backbone root-mean-square fluctuation (in Å) of apo- xCT (orange) and L-cystine-bound xCT upon least squares fit from a 200 ns simulation. Inset - root-mean-square fluctuation monitoring (in Å) of TM8 residues 330-339 (orange: apo- xCT, blue: L-cystine-bound xCT), after fitting the structures on TM8 residue backbones. The fitting on TM8 was done to exclude translation and rotation due to the rest of the structure and to reflect the intrinsic dynamics of TM8. The line widths shown are equal to the bootstrapped (N=1000) error for each residue. When L-cystine is bound, TM8 residues are overall less mobile and more structured indicating increased helicity in the cystine-bound form. **c,** Interaction fingerprint of L-cystine bound to xCT binding site residues, calculated as a fraction of the total simulation time from 3 unbiased simulations (3 x 200 = 600 ns). Cystine interactions per residue can take values > 1 (uncapped), by accounting for interactions from more than 1 atoms per residue with cystine

in a single frame (orange: H-bonds, blue: water bridge, green: salt bridge interactions. Below, analysis of the L-cystine dihedrals colour-coded on the cystine structure shown. Radial plots illustrate the conformational changes of a dihedral over time, and each distribution plot includes a histogram analysis of the dihedrals sampled (bin-width = 10°).

Supplementary Table 1. List of oligonucleotides used in the study.

	Sequence 5′ to 3′
HsSLC7A11_F	cggcggGAGCTCgccaccATGGACTACAAAGACGATGACGACAAGgtcagaaagcctgttgtgtccacc
HsSLC7A11_R	tgaattGTCGACtcataacttatcttcttctggtac
K198A_F	attttcttaaccttttgcGCCctcacagcaattctgata
K198A_R	tatcagaattgctgtgagGGCgcaaaaggttaagaaaat
R135A_F	gtggaactcctcataataGCCcctgcagctactgctgtg
R135A_R	cacagcagtagctgcaggGGCtattatgaggagttccac
R135K_F	gtggaactcctcataataAAGcctgcagctactgctgtg
R135K_R	cacagcagtagctgcaggCTTtattaugaggagttccac
R396A_F	gaatttcctcagttttgccGCCtggctttttattgggctg
R396A_R	cagcccaataaaaagccaGGCggcaaaactgaggaaattc
R396K_F	gaatttcctcagttttgccAAGtggctttttattgggctg
R396K_R	cagcccaataaaaagccaCTTggcaaaactgaggaaattc
R396N_F	gaatttcctcagttttgccAACtggctttttattgggctg
R396N_R	cagcccaataaaaagccaGTTggcaaaactgaggaaattc
Y244A_F	ggctttttattatggaatgGCCgcatatgctggctggttttac
Y244A_R	gtaaaaccagccagcatatgcGGCcattccataataaaaagcc
Y244F_F	ggctttttattatggaatgTTTgcatatgctggctggttttac
Y244F_R	gtaaaaccagccagcatatgcAAAcattccataataaaaagcc
F336Y_F	ccatgaacggtggtgtgtatgctgtctccagg
F336Y_R	cctggagacagcatacacaccaccgttcatgg
F336A_F	ccatgaacggtggtgtggctgctgtctccagg
F336A_R	cctggagacagcagccacaccaccgttcatgg
G334T_F	ctccatgaacggtactgtgtttgctgtctcc
G334T_R	ggagacagcaaacacagtaccgttcatggag
G334A_F	ctccatgaacggtgctgtgtttgctgtctcc
G334A_R	ggagacagcaaacacagcaccgttcatggag
HsSLC3A2_F	cggcggGGTACCgccaccATGCACCATCACCATCACCATCACCATgagctacagcctcctgaagcctcg
HsSLC3A2_R	ccgccgctcgagtcaggccgcgtaggggaagcggag
