# Supplementary material for: Molecular basis for redox control by the human cystine/glutamate antiporter system xc−
Source: Nat Commun. 2021 Dec 8;12:7147. doi: 10.1038/s41467-021-27414-1 (PMC8654953; doi:10.1038/s41467-021-27414-1)
Supplement: Supplementary file 3 — Reporting summary [file 41467_2021_27414_MOESM3_ESM.pdf]

nature research

Corresponding author(s): Joanne.parker@bioch.ox.ac.uk
susan.lea@path.ox.ac.uk
simon.newstead@bioch.ox.ac.uk

Last updated by author(s): Nov 13, 2021

Reporting Summary

Nature Research wishes to improve the reproducibility of the work that we publish. This form provides structure for consistency and transparency in reporting. For further information on Nature Research policies, see our Editorial Policies and the Editorial Policy Checklist.

Statistics

For all statistical analyses, confirm that the following items are present in the figure legend, table legend, main text, or Methods section.

n/a	Confirmed	
☐	☒	The exact sample size (*n*) for each experimental group/condition, given as a discrete number and unit of measurement
☐	☒	A statement on whether measurements were taken from distinct samples or whether the same sample was measured repeatedly
☒	☐	The statistical test(s) used AND whether they are one- or two-sided<br>*Only common tests should be described solely by name; describe more complex techniques in the Methods section.*
☒	☐	A description of all covariates tested
☒	☐	A description of any assumptions or corrections, such as tests of normality and adjustment for multiple comparisons
☐	☒	A full description of the statistical parameters including central tendency (e.g. means) or other basic estimates (e.g. regression coefficient) AND variation (e.g. standard deviation) or associated estimates of uncertainty (e.g. confidence intervals)
☒	☐	For null hypothesis testing, the test statistic (e.g. *F*, *t*, *r*) with confidence intervals, effect sizes, degrees of freedom and *P* value noted<br>*Give P values as exact values whenever suitable.*
☐	☒	For Bayesian analysis, information on the choice of priors and Markov chain Monte Carlo settings
☒	☐	For hierarchical and complex designs, identification of the appropriate level for tests and full reporting of outcomes
☒	☐	Estimates of effect sizes (e.g. Cohen's *d*, Pearson's *r*), indicating how they were calculated

Our web collection on statistics for biologists contains articles on many of the points above.

Software and code

Policy information about availability of computer code

Data collection	Thermofisher EPU used to collect data on microscope
Data analysis	SIMPLE3.0 - available on GitHub https://github.com/hael/SIMPLE3.0 RELION3.1 - published and freely available PHENIX - published and freely available COOT - published and freely available PRISM 9 - commercially available

For manuscripts utilizing custom algorithms or software that are central to the research but not yet described in published literature, software must be made available to editors and reviewers. We strongly encourage code deposition in a community repository (e.g. GitHub). See the Nature Research guidelines for submitting code & software for further information.

Data

Policy information about availability of data

All manuscripts must include a data availability statement. This statement should provide the following information, where applicable:
- Accession codes, unique identifiers, or web links for publicly available datasets
- A list of figures that have associated raw data
- A description of any restrictions on data availability

Cryo-EM volumes and atomic models have been deposited to the EMDB (accession codes EMD-13267, EMD-13266) and PDB (accession codes 7P9V and 7P9U) respectively.

Field-specific reporting

Please select the one below that is the best fit for your research. If you are not sure, read the appropriate sections before making your selection.

☒ Life sciences ☐ Behavioural & social sciences ☐ Ecological, evolutionary & environmental sciences

For a reference copy of the document with all sections, see nature.com/documents/nr-reporting-summary-flat.pdf

Life sciences study design

All studies must disclose on these points even when the disclosure is negative.

Sample size	Sample sizes chosen based on standard procedures for biochemical assays and computational analysis to ascertain accurate values for the data shown. Statistical methods were not used to determine sample size, but were used to calculate standard deviations.
Data exclusions	No data were excluded.
Replication	All assays were repeated a minimum of three times and were reproducible.
Randomization	Samples were not randomized for the experiments as this was not applicable for the biochemical assays described in our study.
Blinding	No blinding was applied to the data generated in our study, as this is not applicable to the types of biochemical or structural data generated.

Reporting for specific materials, systems and methods

We require information from authors about some types of materials, experimental systems and methods used in many studies. Here, indicate whether each material, system or method listed is relevant to your study. If you are not sure if a list item applies to your research, read the appropriate section before selecting a response.

Materials & experimental systems

n/a	Involved in the study	
☒	☐	Antibodies
☐	☒	Eukaryotic cell lines
☒	☐	Palaeontology and archaeology
☒	☐	Animals and other organisms
☒	☐	Human research participants
☒	☐	Clinical data
☒	☐	Dual use research of concern

Methods

n/a	Involved in the study	
☒	☐	ChIP-seq
☒	☐	Flow cytometry
☒	☐	MRI-based neuroimaging

Eukaryotic cell lines

Policy information about cell lines

Cell line source(s)	HEK293F purchased from ThermoFisher; HeLa derived cell line was obtained internally within the University. Originally sourced from Invitrogen Life Technologies.
Authentication	The cell lines were not authenticated.
Mycoplasma contamination	The cell lines were not tested for mycoplasma contamination.
Commonly misidentified lines (See ICLAC register)	None.
